# The Relation Between Violence and Suicidality in Mexico: The Impact of Different Types of Violence in Suicidal Behaviour Detected by a Massive Mental Health Screening App (MeMind)

**DOI:** 10.3390/bs15081117

**Published:** 2025-08-18

**Authors:** Cristian Antonio Molina-Pizarro, Olatz Lopez-Fernandez, Paula Villasante-Soriano, Ismael Martinez-Nicolas, Fuensanta Aroca-Bisquert, Pablo Méndez-Bustos, Lucas Giner, Enrique Baca-García

**Affiliations:** 1 Instituto de Salud Mental del Gobierno de Yucatán (Institute of Mental Health of the Government of Yucatan), Merida 97000, Mexico; molina_cripiz@gva.es; 2 Department of Behavioural Sciences Methodology, Faculty of Psychology, Universidad Nacional de Educación a Distancia (UNED), Moncloa-Aravaca, 28040 Madrid, Spain; 3 Department of Psychiatry, University Hospital Jimenez Diaz Foundation, 28040 Madrid, Spain; pvillasante@uoc.edu; 4 Faculty of Life Sciences, Universidad Católica San Antonio de Murcia (UCAM), 30107 Guadalupe, Spain; ismael.martinez2@carm.es; 5 Sistema Español de Notificación en Seguridad en Anestesia y Reanimación (SENSAR), IDEhA Simulatio Centre, Fundación Alcorcon University Hospital, 28922 Alcorcon, Spain; 6 Instituto de Matemáticas, Universidad Nacional Autónoma de México (UNAM), Mexico City 14610, Mexico; fuen@im.unam.mx; 7 Department of Psychology, Universidad Católica del Maule, Talca 3460000, Maule, Chile; pmendez@ucm.cl; 8 Department of Psychiatry, Universidad de Sevilla, 41009 Seville, Spain; lginer@us.es; 9 Department of Psychiatry, Universidad Autónoma de Madrid, 28029 Madrid, Spain; 10 Department of Psychiatry, Hospital Rey Juan Carlos, 28933 Móstoles, Spain; 11 Department of Psychiatry, Central Hospital de Villalba, 28400 Villalba, Spain; 12 Department of Psychiatry, University Hospital Infanta Elena, 28034 Valdemoro, Spain; 13 CIBERSAM, Research Group CB/07/09/0025, 28007 Madrid, Spain

**Keywords:** violence at home, self-injury, suicide ideation, suicide attempt, smartphone screening, Mexico

## Abstract

The construct of violence has scarcely been researched in relation to suicidality in Mexico. The aim of the present study was to explore the role of different types of violence (e.g., violence at home, non-suicidal self-injury) in suicidal behaviour (e.g., suicidal ideation, suicidal acts) of the citizens of the Yucatan State using a massive online screening approach through a smartphone application. A prospective cohort study design was undertaken during 2022 including 32,531 Mexican participants aged between 15 and 80 years old, which constituted the second wave of the SmartScreen project, through the TEDUCA survey. We selected as the main variables violence at home, non-suicidal self-injuries, and suicidal behaviour (Columbia—Suicide Severity Rating Scale; C-SSRS). A set of univariate regression analyses was performed for the entire sample connecting the variables with the C-SSRS. Subsequently, a multiple linear regression model was used. The model explained a significant portion of the variance in C-SSRS scores (*R*^2^adj = 0.3227) indicating the following as predictors affecting suicidality: perceived violence at home, followed by a history of NSSI, and previous mental health service attendance, among other associations between the sociodemographic predictors and suicidal behaviour. Significant interactions between perceived violence and NSSI history were also found. In conclusion, our study highlights the significant role of perceived violence at home and NSSI history in shaping suicide risk understood as the ideation and intention of suicidality among Yucatan citizens. The interaction between these factors and sociodemographic variables such as gender and age underscore the complex nature of suicide risk.

## 1. Introduction

The construct of violence has been explicitly identified as a significant public health issue, as it can harm the physical and mental well-being of individuals (Rutherford et al., 2007). At present, the framework of the United Nations’ 2030 Agenda for Sustainable Developments has recognized violence as a critical topic, and different proposals have targeted goals related to violence prevention to work on global public health actions worldwide (Lee et al., 2016b).

According to the World Report on Violence and Health (WRVH) (World Health Organization [WHO], 2004), violence is defined as the intentional use of force or power, threatened or actual, against oneself, another person, or against a group or community, whilst suicide refers to the deliberate act of ending one’s own life, and suicidal behaviour pertains to non-fatal suicidal thoughts and actions, such as suicide ideation, planning, and attempts, following the terminology established by Posner et al. (2007) and Silverman et al. (2007a, 2007b). Violence can occur in four modes (i.e., physical, sexual, psychological, and deprivation), and three sub-types have been described based on the victim–perpetrator relationship: (i) self-directed violence (i.e., self-injury, self-abuse, and suicide); (ii) interpersonal violence at the family level (e.g., child maltreatment, intimate partner violence (IPV), or elder abuse); and (iii) the non-family level (e.g., community violence perpetrated by an acquaintance or stranger). Studies show that suicidal behaviour is usually associated with violence types (Centers for Disease Control and Prevention [CDC], 2016; Decker et al., 2018), such as violence at home (Brown & Seals, 2019) or self-injury (Liu et al., 2022). Angelakis et al. (2020) quantified the association between the core types of childhood maltreatment and suicide behaviour in children and young adults, all of which induced 2.5-fold greater odds of suicide ideation, except for child sexual abuse (CSA), which caused a 4.0-fold increase in suicide plans.

Conceptual models of suicidal behaviour have identified a history of violence as a critical risk factor. A systematic and narrative review conducted by Díaz-Oliván et al. (2021) has outlined, from theoretical models of suicidal behaviour, a set of variables associated with various types of violence: (i) self-directed violence (e.g., feelings of defeat, entrapment, aggression, and self-injury); (ii) family-related violence (e.g., neglect, CSA and physical abuse); and (iii) non-family violence (e.g., social exclusion or traumatic life events). Although self-injuries is a classic risk factor, CSA emerged as the most prevalent factor linked to suicidality according to the theoretical models.

In the American continent, violence is one of the leading sources of morbidity and mortality (Decker et al., 2018). It is noteworthy that empirical studies in the United States of America (USA) have reinforced the relationship between violence and suicide in which the interplay exists in both directions, with self-directed and other-directed violence serving as underlying factors causing, at least, suicidal ideation. For instance, North American cross-sectional studies indicate that exposure to violence acts as a risk factor for future suicidal behaviour, with self-directed and interpersonal violence frequently occurring in conjunction. Research in the USA has demonstrated that the impact of exposure to violence on suicidal ideation is fully mediated and stronger in females and adolescents (Farrell & Zimmerman, 2018). Similarly, in longitudinal studies, a reciprocal relationship between violence and suicidality has been observed from adolescence into adulthood, especially in males during early adulthood (Van Dulmen et al., 2013).

Nevertheless, most of the global violence occurs in regions such as Latin American countries (Lee et al., 2016a). Indeed, a surge in the incidence of violent deaths among young individuals, particularly males, has been noted in Mexico (González-Pérez et al., 2017) where both violence and suicidal behaviour are more prevalent compared to other regions (World Health Organization [WHO], 2021). However, the relationship between different types of violence and suicidal behaviour has been scarcely investigated in Mexico, although previous research has studied the prevalence and associated psychological factors of attempted suicide in a sample of Mexican adolescents (Valdez-Santiago et al., 2018a). Exposure to interpersonal and community violence is strongly linked to adverse mental health outcomes among Mexican adolescents, including heightened risks of anxiety, depression, and suicidal behaviour. Experiences such as domestic violence, school bullying, and exposure to organized crime contribute to a recurring cycle of trauma that frequently goes unaddressed due to systemic deficiencies in institutional support for youth (Valdez-Santiago et al., 2020). Y. Cruz-Manrique et al. (2021) found that school-based victimization, compounded by a lack of family support, was a strong predictor of suicidal ideation among adolescents in Yucatán.

Comprehending the magnitude of the connection between violence types and suicidal behaviour is crucial for understanding the relationship between both to develop effective interventions and policies to address the problem contextualized to specific population groups. Consequently, the main aim of the present study is to explore the role of different types of violence, such as violence at home, non-suicidal self-injury, and a lack of well-being, in the suicidal behaviour (i.e., suicidal ideation and suicidal acts) of the Yucatan State (Mexico) general population using massive online screening approach.

## 2. Methods

### 2.1. Design

This investigation was conducted under a quantitative, positivist paradigm using a cross-sectional survey design. Data were collected in a single wave in 2023 via the SmartScreen project’s platform (MeMind app), targeting residents of the State of Yucatán, Mexico.

### 2.2. Participants

This study included 32,531 Mexican participants from the general population in the State of Yucatan, from which 16,267 (50.0%) were women. In total, 192 (0.6%) participants identified their gender as non-binary or as not falling within the categories of woman or man. The age range was 15 to 80 years old.

Our inclusion criteria were as follows: (1) age 15 years or above with enrolment at a participating institution (e.g., education centres, organizations); (2) the ability to use their smartphones; (3) the ability to understand the nature, purpose, and methodology of the study; and (4) participation following express informed consent granted in the app. The exclusion criteria were as follows: (1) students protected by law (e.g., conservatorship) and (2) individuals deprived of liberty (e.g., prisoners).

### 2.3. Instruments

Our main variables were violence at home (VH), non-suicidal self-injuries (NNSI), and suicidal behaviour (SB). These were derived from reported theoretical models’ systematic review and empirical studies, which are described as follows:

The variable violence at home (VH) was assessed using a 5-point Likert scale ranging from “unusual” to “very frequent.” This ordinal measure captured the participant’s perception of the frequency of violence currently occurring within their home environment, specifically focusing on intrafamilial violence (e.g., “Do you think violence at home or in the family is…”).

NNSI was an item about NSSI adapted from DMS 5-2: Have you ever intentionally injured yourself without the intent to take your own life (e.g., cut yourself, hit yourself, stuck yourself with needles, burned yourself, scratched your skin with force, etc.)?

SB was measured through the Columbia—Suicide Severity Rating Scale (C-SSRS) (Mundt et al., 2013; Posner et al., 2011). It includes seven yes/no items regarding the participant’s risk of suicidality. The Spanish version used (Al-Halabí et al., 2016) showed good internal consistency (Cronbach’s α = 0.81) and has also been used in a previous study with another cohort of participants (Martínez-Nicolás et al., 2022b).

These mental health tests have demonstrated adequate validity as a screening tool in several countries including Mexico. Additionally, we employed the following variables: previous mental health service attendance, NSSI history, gender, age, occupation, number of household members, and marital status as secondary variables.

For more information regarding the selection of the tests and items for the Smart-Screen Project in Mexico, please see Arenas-Castañeda et al. (2020).

### 2.4. Procedure

The data were retrieved in 2023 from the second wave of the SmartScreen project, through the TEDUCA survey (Martínez-Nicolás et al., 2022b), through a large suicide risk and NSSI behaviour assessment. The TEDUCA is an ongoing online survey through a mHealth smartphone app named MeMind (Berrouiguet et al., 2015) and, according to SmartScreen’s protocol (Martínez-Nicolás et al., 2022a), its primary purpose is to identify the correlates of potential unhealthy populations concerning suicide. For all the details about the SmartScreen protocol survey, please see Arenas-Castañeda et al. (2020). Ethical approval (002/2019) was granted by the Ethics Review Board of the ‘Hospital Psiquiátrico Yucatán’, Yucatan (Mexico). This protocol has been registered in ClinicalTrials.gov. as the SmartScreen Project (Arenas-Castañeda et al., 2020).

Answers to MeMind questions compiled within the app were uploaded to a secure web server. Usernames and personal data were pseudonymized with a code. Data were encrypted using AES-256 algorithms and 256-bit keys protected by a professional key management infrastructure externally audited.

### 2.5. Analysis

Descriptive and exploratory analyses were performed to describe participant characteristics. After that, univariate regressions were conducted to determine if the selected variables (violence at home, NSSI history, gender, age, occupation, number of household members, and marital status) were significant in the total score of the C-SSRS test following the purposeful selection process, whereby any variable with a statistically significant result in the univariate analysis was selected as a candidate for the multivariate analysis (Bursac et al., 2008).

Upon confirming that all variables were significant in the univariate regression analyses, including the interaction between violence at home and NSSI, a multiple linear regression model was constructed to analyse the relationship between the predictor variables, the interaction and the total score of the C-SSRS test.

Statistical analyses were conducted using R statistical software. R is available as an open access software under the terms of the GNU General Public License (Lafaye de Micheaux et al., 2013).

## 3. Results

The descriptive statistics are reported in Table 1. Significant differences across genders were observed across all variables analysed (*p* < 0.001). Gender was balanced (50% women), and among the age groups studied, those situated between the late adolescence and middle-aged adulthood categories were more prevalent than in the elderly age group (1%). The majority of the participants (90%) used to live in homes with a small number of cohabitants (from two to six persons), in which almost half of the sample was single and did not have a child under their care. Regarding current labour activity, more than half of the sample was active as a third were high school and undergraduate students. Concerning the aggressive variables, only a tenth (10%) committed NSSI behaviours, almost a fifth (17%) had some type of suicide risk, and more than a half of the sample (61%) reported to have experienced violence at home.

Table 2 presents the outcomes of multiple linear regression (MLR) applied to the training data. Significance was found in all model parameters concerning the effectiveness of treatment (*p* < 0.0001).

The model demonstrated good fit, explaining a significant portion of variance in C-SSRS scores.

Perceived violence at home was a significant predictor, with individuals perceiving very frequent (*b* = 0.103, *p* < 0.001), frequent (*b* = 0.096, *p* < 0.001), and somewhat frequent violence (*b* = 0.087, *p* < 0.001) compared to those who did not perceive violence at home (Table 2).

A history of NSSI was strongly associated with higher C-SSRS scores (*b* = 1.573, *p* < 0.001). Gender also played a role, with women having slightly higher scores compared to men (*b* = 0.035, *p* = 0.013) and individuals identifying as non-binary having significantly higher scores (*b* = 1.094, *p* < 0.001).

Previous mental health service attendance was another significant predictor, with individuals who had previously accessed mental health services having higher C-SSRS scores (*b* = 0.301, *p* < 0.001).

Regarding occupation, those who were unemployed had higher C-SSRS scores compared to retired individuals (*b* = 0.812, *p* < 0.001). Homemakers showed a marginal increase in scores (*b* = 0.268, *p* = 0.094), while other occupations did not show significant differences.

The number of household members was inversely related to C-SSRS scores, with individuals living with 1–3 (*b* = −0.161, *p* < 0.001), 4–6 (*b* = −0.169, *p* < 0.001) or more than 6 members (*b* = −0.150, *p* < 0.001) having lower scores compared to those living by their own. Marital status was significant, with individuals in a couple (*b* = 0.096, *p* < 0.001) showing higher scores.

Age showed a significant negative relationship with C-SSRS scores. Younger individuals (18–25 years) had lower scores (*b* = −0.139, *p* < 0.001) compared to individuals between 15 and 18 years old, and the scores continued to decrease significantly across the older age groups (25–35, 35–50, 50–65, and 65+).

Significant interactions between perceived violence and NSSI history were found, indicating that the relationship between violence perception and C-SSRS scores is moderated by NSSI history. Individuals perceiving very frequent violence combined with a history of NSSI (*b* = 0.569, *p* < 0.001), frequent violence combined with NSSI (*b* = 0.329, *p* < 0.001), somewhat frequent violence combined with NSSI (*b* = 0.435, *p* < 0.001), and an uncertain frequency of violence combined with NSSI (*b* = 0.198, *p* = 0.048) had significantly higher C-SSRS scores compared to non-perceived violence at home combined with no NSSI history.

## 4. Discussion

Our study investigated the associations between perceived violence at home, NSSI history, and suicide risk among individuals assessed using the Columbia—Suicide Severity Rating Scale. The main findings underscore the significance of perceived violence at home and NSSI history as pivotal factors influencing suicide risk scores. However, gender and sociodemographic characteristics such as age, and occupation were found also as meaningful predictors. Each of these factors contributes uniquely to the overall risk. Hence, our results contribute to a better understanding of the multifaceted nature of suicide risk in relation to violence types, necessitating a comprehensive approach in both assessment and intervention strategies.

Specifically, the multivariate analysis revealed that the perception of frequent violence at home significantly increased C-SSRS scores, with a gradient effect observed across categories from frequent to very frequent violence. This trend remained significant after adjusting for sociodemographic variables such as gender and age. Furthermore, a history of NSSI was strongly associated with elevated C-SSRS scores, particularly when interacting with the perception of violence at home, as also shown in females in previous studies.

The impact of interpersonal violence on suicide risk has been well documented. MacIsaac et al. (2017) systematically reviewed the association between exposure to interpersonal violence and completed suicide among women, either as victims or perpetrators, finding a connection with no clear association among mental health factors. Similarly, Angelakis et al. (2020) conducted a systematic review and meta-analysis, quantifying the association between various forms of childhood maltreatment and suicide behaviour in young individuals. They found a 2.5-fold greater risk for suicide ideation, with child sexual abuse leading to a 4.0-fold increase in suicide plans. Moreover, a global study on intimate partner violence and mental health outcomes in women revealed increased odds for suicidality (OR = 2.17–5.52), further demonstrating the serious mental health consequences of violence exposure (R. G. White et al., 2024). A. Clarke et al. (2019) also highlighted the broader context of NSSI, encompassing various forms of violence and their connections to suicidality.

Hence, our findings in the present study applied to Mexico are consistent with previous research highlighting the link between violence and suicide risk. Studies have repeatedly demonstrated that individuals exposed to domestic violence are at a heightened risk for developing suicidal ideation and behaviours, particularly when compounded by a history of NSSI. Moreover, the gender differences in suicide risk observed in our study align with global patterns that suggest women may experience more severe mental health impacts from domestic violence.

Indeed, gender seems to play a notable role in suicide risk. Valdez-Santiago et al. (2018b) explored attempted suicide among Mexican adolescents, finding a 2.7% prevalence, with women being significantly more at risk than men. Women and non-binary individuals had significantly higher C-SSRS scores compared to men when controlling for the perception of violence and NSSI history (Mendoza-Pérez et al., 2021; Valdez-Santiago et al., 2018a). Interestingly, the interactions between gender and perceived violence suggest that women and non-binary individuals may experience an amplified suicide risk in environments of domestic violence compared to men.

Additionally, adolescents who had experienced violence-related health damage in the previous year had over four times the risk of engaging in suicide attempts. Indeed, younger age groups and individuals who had previously sought mental health services demonstrated higher suicide risk scores, emphasizing the importance of early mental health intervention in this population (Liu et al., 2022). Social factors should be considered in future locally adapted population-based interventions. For instance, A. L. Cruz-Manrique et al. (2021) found that school victimization and a lack of family support were important predictors of suicidal ideation among adolescents in the State of Yucatan. These findings reflect the need for considering younger, female and minorities populations (e.g., Mexican homosexual and bisexual men). They also add to the broader literature on the cumulative effects of adverse childhood experiences on long-term mental health outcomes (Anda et al., 2006), which require in turn a dedicated, priority effort to be addressed.

### 4.1. Clinical and Policy Implications

The findings from this study have important clinical implications, particularly in the context of screening and intervention for suicide risk. Given the strong association between perceived violence and NSSI, clinicians should prioritize comprehensive assessments that include questions about domestic violence and NSSI behaviours when evaluating suicide risk.

The gendered disparities found in this study reflect broader social and structural inequalities that disproportionately affect women and sexual and gender minorities in Mexico (Mendoza-Pérez et al., 2021; Valdez-Santiago et al., 2018b). These groups often face compounded forms of violence, including gender-based discrimination, limited access to healthcare, and social stigmatization (A. L. Cruz-Manrique et al., 2021; S. J. White et al., 2024), all of which may exacerbate suicide risk. Previous studies have shown that non-binary and LGBTQ+ individuals in Mexico experience higher levels of psychological distress and suicidal ideation due to these intersecting vulnerabilities (Angelakis et al., 2020; Anda et al., 2006). As such, interventions must go beyond clinical assessment to incorporate gender-sensitive and culturally responsive approaches—ensuring that prevention and care strategies address the specific needs, vulnerabilities, and strengths of diverse gender populations (S. Clarke et al., 2019; MacIsaac et al., 2017).

This is particularly crucial for younger individuals and those with a history of mental health service use (Dube et al., 2001). Furthermore, interventions should be gender-sensitive, as women and non-binary individuals appear to be disproportionately affected by violence in relation to suicide risk.

From a policy perspective, our results support the need for robust domestic violence prevention programmes and the integration of mental health services for individuals exposed to violence. Public health initiatives aimed at reducing domestic violence and providing timely mental health support may significantly mitigate the suicide risk among vulnerable populations (Krug et al., 2002).

### 4.2. Limitations

One of the main limitations of our study is its reliance on self-reported measures of violence and suicide risk, which may be subject to social desirability bias. Additionally, the cross-sectional design limits our ability to infer causal relationships between perceived violence and suicide risk. Future longitudinal studies are needed to establish the temporal sequence between these variables. However, the large sample size and comprehensive statistical analysis employed in this study are notable strengths that enhance the reliability of our findings (Friedman & Sudak, 2019).

## 5. Conclusions

In conclusion, our study highlights the significant role of perceived violence at home and NSSI history in shaping suicide risk as measured by the C-SSRS. The interaction between these factors and sociodemographic variables such as gender and age underscore the complex nature of suicide risk. Future research should focus on longitudinal analyses to better understand the causal pathways involved. Clinicians and policymakers should prioritize the early identification of risks and intervention for individuals exposed to violence and those with a history of NSSI to reduce the burden of suicide in these populations.

## Figures and Tables

**Table 1:** Descriptive statistics and sociodemographics.

		Overall	Man	Woman	Other	*p*-Value
Age	<18	5083 (15.6)	2188 (13.6)	2802 (17.2)	93 (48.4)	<0.001
	18–25	6791 (20.9)	3512 (21.9)	3228 (19.8)	51 (26.6)	
	25–35	6862 (21.1)	3281 (20.4)	3556 (21.9)	25 (13.0)	
	35–50	9875 (30.4)	5021 (31.2)	4837 (29.7)	17 (8.9)	
	50–65	3623 (11.1)	1873 (11.7)	1745 (10.7)	5 (2.6)	
	>65	297 (0.9)	197 (1.2)	99 (0.6)	1 (0.5)	
Marital status	Single	13,788 (52.1)	6376 (49.0)	7300 (54.8)	112 (78.9)	<0.001
	Common-law partner	2279 (8.6)	1079 (8.3)	1188 (8.9)	12 (8.5)	
	Married	8738 (33.0)	4942 (38.0)	3782 (28.4)	14 (9.9)	
	Separated/divorced	1448 (5.5)	545 (4.2)	899 (6.7)	4 (2.8)	
	Widowed	215 (0.8)	64 (0.5)	151 (1.1)	0 (0.0)	
Current activity	Retired	86 (0.3)	48 (0.3)	37 (0.3)	1 (0.6)	<0.001
	Active	18,906 (65.1)	9654 (67.5)	9210 (63.2)	42 (27.3)	
	Homemaker	560 (1.9)	110 (0.8)	445 (3.1)	5 (3.2)	
	Student	8462 (29.2)	4057 (28.4)	4309 (29.6)	96 (62.3)	
	Unemployed	144 (0.5)	62 (0.4)	77 (0.5)	5 (3.2)	
	Underemployed	870 (3.0)	379 (2.6)	486 (3.3)	5 (3.2)	
Number of cohabitants	Alone	1127 (4.3)	614 (4.7)	504 (3.8)	9 (6.2)	<0.001
	1–3	12,581 (47.5)	6045 (46.5)	6477 (48.6)	59 (41.0)	
	4–6	11,133 (42.1)	5571 (42.8)	5500 (41.3)	62 (43.1)	
	More than 6	1625 (6.1)	777 (6.0)	834 (6.3)	14 (9.7)	
Violence at home	Non-existent	10,645 (37.1)	5673 (40.1)	4941 (34.2)	31 (20.9)	<0.001
	Very frequent	3319 (11.6)	1075 (7.6)	2229 (15.4)	15 (10.1)	
	Frequent	5571 (19.4)	2449 (17.3)	3076 (21.3)	46 (31.1)	
	Not very frequent	7870 (27.4)	4258 (30.1)	3571 (24.7)	41 (27.7)	
	Do not know	1318 (4.6)	690 (4.9)	613 (4.2)	15 (10.1)	
NSSI	No	26,390 (89.6)	13,667 (94.3)	12,652 (85.4)	71 (46.1)	<0.001
	Yes	3066 (10.4)	827 (5.7)	2156 (14.6)	83 (53.9)	
CSSRS		5.86 (2.20)	5.71 (2.07)	5.99 (2.27)	6.97 (4.17)	<0.001
	TOTAL	32,531	16,072 (49.4)	16,267 (50.0)	192 (0.6)	

**Table 2:** Multiple linear regression (MLR).

Predictor	β	95% CI	*p*-Value
Intercept	6.57 **	[6.26, 6.88]	<0.01
Had sought any mental health services (ref: no)			
Yes	0.30 **	[0.27, 0.33]	<0.01
Violence at home (ref: inexistent violence)			
Very frequent	0.10 **	[0.05, 0.15]	<0.01
Frequent	0.10 **	[0.06, 0.14]	<0.01
Not frequent	0.09 **	[0.05, 0.12]	<0.01
Does not know how often violence occurs at home	0.07	[−0.00, 0.14]	0.06
Had intentionally inflicted NSSI before (ref: no)			
Yes	1.57 **	[1.49, 1.66]	<0.01
Gender (ref: Man)			
Woman	0.04 *	[0.01, 0.06]	0.01
Other	1.09 **	[0.91, 1.28]	<0.01
Current activity (ref: retired)			
Active	−0.13	[−0.43, 0.16]	0.38
Homemaker	0.27	[−0.05, 0.58]	0.09
Student	0.00	[−0.30, 0.30]	0.99
Unemployed	0.81 **	[0.46, 1.16]	<0.01
Underemployed (temporary)	−0.03	[−0.34, 0.27]	0.83
Household members (ref: 0)			
1–3	−0.16 **	[−0.23, −0.09]	<0.01
4–6	−0.17 **	[−0.24, −0.10]	<0.01
More than 6	−0.15 **	[−0.24, −0.06]	<0.01
Family status			<0.01
Domestic partner	0.10 **	[0.05, 0.15]	<0.01
Married	0.00	[−0.04, 0.04]	0.87
Divorced/separated	0.06	[−0.00, 0.13]	0.06
Widowed	0.05	[−0.10, 0.20]	0.53
Age group (ref: <18)			
18–25	−0.14 **	[−0.18, −0.09]	<0.01
25–35	−0.29 **	[−0.35, −0.22]	<0.01
35–50	−0.34 **	[−0.41, −0.28]	<0.01
50–65	−0.34 **	[−0.42, −0.27]	<0.01
>65	−0.45 **	[−0.63, −0.28]	<0.01
Violence at home: had intentionally inflicted NSSI before (ref: Inexistent violence: No)			<0.01
Very frequent: Yes	0.57 **	[0.43, 0.71]	<0.01
Frequent: Yes	0.33 **	[0.21, 0.45]	<0.01
Not frequent: Yes	0.44 **	[0.32, 0.55]	<0.01
Does not know: Yes	0.20 *	[0.00, 0.39]	0.05

* *p* < 0.1; ** *p* < 0.01.

## Data Availability

The datasets used and/or during the current study are available from the corresponding author on reasonable request.

## References

[B1-behavsci-15-01117] Al-Halabí S., Sáiz P. A., Burón P., Garrido M., Benabarre A., Jiménez E., Cervilla J., Navarrete M. I., Díaz-Mesa E. M., García-Álvarez L., Muñiz J., Posner K., Oquendo M. A., García-Portilla M. P., Bobes J. (2016). Validation of a Spanish version of the Columbia-suicide severity rating scale (C-SSRS) [Validación de la versión en Español de la Columbia-suicide severity rating scale (escala columbia para evaluar el riesgo de suicidio)]. Revista de Psiquiatria y Salud Mental.

[B2-behavsci-15-01117] Anda R. F., Felitti V. J., Bremner J. D., Walker J. D., Whitfield C., Perry B. D., Dube S. R., Giles W. H. (2006). The enduring effects of abuse and related adverse experiences in childhood. European Archives of Psychiatry and Clinical Neuroscience.

[B3-behavsci-15-01117] Angelakis I., Austin J. L., Gooding P. (2020). Association of childhood maltreatment with suicide behaviors among young people: A systematic review and meta-analysis. JAMA Network Open.

[B4-behavsci-15-01117] Arenas-Castañeda P. E., Aroca Bisquert F., Martinez-Nicolas I., Castillo Espíndola L. A., Barahona I., Maya-Hernández C., Lavana Hernández M. M., Manrique Mirón P. C., Alvarado Barrera D. G., Treviño Aguilar E., Barrios Núñez A., De Jesus Carlos G., Vildosola Garcés A., Flores Mercado J., Barrigon M. L., Artes A., de Leon S., Molina-Pizarro C. A., Rosado Franco A., Baca-Garcia E. (2020). Universal mental health screening with a focus on suicidal behaviour using smartphones in a Mexican rural community: Protocol for the SMART-SCREEN population-based survey. BMJ Open.

[B5-behavsci-15-01117] Berrouiguet S., Courtet P., Perez-Rodriguez M., Oquendo M., Baca-Garcia E. (2015). The Memind project: A new web-based mental health tracker designed for clinical management and research. European Psychiatry.

[B6-behavsci-15-01117] Brown S., Seals J. (2019). Intimate partner problems and suicide: Are we missing the violence?. Journal of Injury & Violence Research.

[B7-behavsci-15-01117] Bursac Z., Gauss C. H., Williams D. K., Hosmer D. W. (2008). Purposeful selection of variables in logistic regression. Source Code for Biology and Medicine.

[B8-behavsci-15-01117] Centers for Disease Control and Prevention (CDC) (2016). Preventing multiple forms of violence: A strategic vision for connecting the dots.

[B9-behavsci-15-01117] Clarke A., Patalay P., Allen E., Knight L., Naker D., Devries K. (2019). Patterns and predictors of self-harm in poor urban young adolescents in sub-Saharan Africa: A prospective cohort study. BMC Public Health.

[B10-behavsci-15-01117] Clarke S., Allerhand L. A., Berk M. S. (2019). Recent advances in understanding and managing self-harm in adolescents. F1000Research.

[B11-behavsci-15-01117] Cruz-Manrique A. L., Martínez-Valle A., Medina-Mora M. E. (2021). Prevalencia e impacto de la victimización escolar en la ideación suicida de adolescentes en Yucatán. Salud Mental.

[B12-behavsci-15-01117] Cruz-Manrique Y., Olaizola J. H., Cortés-Ayala L., Malvaceda-Espinoza E. (2021). Effect of violence and school victimization on suicidal ideation in Mexican adolescents. International Journal of Psychological Research.

[B13-behavsci-15-01117] Decker M. R., Wilcox H. C., Holliday C. N., Webster D. W. (2018). An integrated public health approach to interpersonal violence and suicide prevention and response. Public Health Reports (Washington, D.C.: 1974).

[B14-behavsci-15-01117] Díaz-Oliván I., Porras-Segovia A., Barrigón M. L., Jiménez-Muñoz L., Baca-García E. (2021). Theoretical models of suicidal behaviour: A systematic review and narrative synthesis. The European Journal of Psychiatry.

[B15-behavsci-15-01117] Dube S. R., Anda R. F., Felitti V. J., Chapman D. P., Williamson D. F., Giles W. H. (2001). Childhood abuse, household dysfunction, and the risk of attempted suicide throughout the life span: Findings from the adverse childhood experiences study. JAMA.

[B16-behavsci-15-01117] Farrell C., Zimmerman G. M. (2018). Violent lives: Pathways linking exposure to violence to suicidal behavior in a national sample. Archives of Suicide Research: Official Journal of the International Academy for Suicide Research.

[B17-behavsci-15-01117] Friedman R. A., Sudak D. M. (2019). Suicide prevention: A public health imperative. Journal of the American Medical Association.

[B18-behavsci-15-01117] González-Pérez G. J., Vega-López M. G., Souza E. R., Pinto L. W. (2017). Violence deaths and its impact on life expectancy: A comparison between Mexico and Brazil [Mortalidad por violencias y su impacto en la esperanza de vida: Una comparación entre México y Brasil]. Ciencia & Saude Coletiva.

[B19-behavsci-15-01117] Krug E. G., Dahlberg L. L., Mercy J. A., Zwi A. B., Lozano R. (2002). World report on violence and health.

[B20-behavsci-15-01117] Lafaye de Micheaux P., Drouilhet R., Liquet B. (2013). The R software.

[B21-behavsci-15-01117] Lee B. X., Gilligan J., Kaaya S. F., Schuder K. K. (2016a). Violence and health: Implications of the 2030 agenda for South-North collaboration. International Journal of Public Health.

[B22-behavsci-15-01117] Lee B. X., Kjaerulf F., Turner S., Cohen L., Donnelly P. D., Muggah R., Davis R., Realini A., Kieselbach B., MacGregor L. S., Waller I., Gordon R., Moloney-Kitts M., Lee G., Gilligan J. (2016b). Transforming our world: Implementing the 2030 agenda through sustainable development goal indicators. Journal of Public Health Policy.

[B23-behavsci-15-01117] Liu R. T., Walsh R. F. L., Sheehan A. E., Cheek S. M., Sanzari C. M. (2022). Prevalence and correlates of suicide and nonsuicidal self-injury in children: A systematic review and meta-analysis. JAMA Psychiatry.

[B24-behavsci-15-01117] MacIsaac M. B., Bugeja L. C., Jelinek G. A. (2017). The association between exposure to interpersonal violence and suicide among women: A systematic review. Australian and New Zealand Journal of Public Health.

[B25-behavsci-15-01117] Martínez-Nicolás I., Arenas Castañeda P. E., Molina-Pizarro C. A., Rosado Franco A., Maya-Hernández C., Barahona I., Martínez-Alés G., Aroca Bisquert F., Baca-García E., Barrigón M. L. (2022a). Impact of depression on anxiety, well-being, and suicidality in Mexican adolescent and young adult students from Mexico city: A mental health screening using smartphones. The Journal of Clinical Psychiatry.

[B26-behavsci-15-01117] Martínez-Nicolás I., Molina-Pizarro C. A., Franco A. R., Arenas Castañeda P. E., Maya C., Barahona I., Martínez-Alés G., Bisquert F. A., Delgado-Gomez D., Dervic K., Lopez-Fernandez O., Baca-García E., Barrigón M. L. (2022b). What seems to explain suicidality in yucatan mexican young adults? Findings from an app-based mental health screening test using the smart-screen protocol. Current Psychology: A Journal for Diverse Perspectives on Diverse Psychological Issues.

[B27-behavsci-15-01117] Mendoza-Pérez J. C., González-Forteza C., Borges G., Benjet C. (2021). Suicide risk and associated factors in Mexican LGBTQ+ youth. Revista Latinoamericana de Psicología.

[B28-behavsci-15-01117] Mundt J. C., Greist J. H., Jefferson J. W., Federico M., Mann J. J., Posner K. (2013). Prediction of suicidal behavior in clinical research by lifetime suicidal ideation and behavior ascertained by the electronic Columbia-suicide severity rating scale. The Journal of Clinical Psychiatry.

[B29-behavsci-15-01117] Posner K., Brown G. K., Stanley B., Brent D. A., Yershova K. V., Oquendo M. A., Currier G. W., Melvin G. A., Greenhill L., Shen S., Mann J. J. (2011). The Columbia-suicide severity rating scale: Initial validity and internal consistency findings from three multisite studies with adolescents and adults. The American Journal of Psychiatry.

[B30-behavsci-15-01117] Posner K., Oquendo M. A., Gould M., Stanley B., Davies M. (2007). Columbia classification algorithm of suicide assessment (C-CASA): Classification of suicidal events in the FDA’s pediatric suicidal risk analysis of antidepressants. The American Journal of Psychiatry.

[B31-behavsci-15-01117] Rutherford A., Zwi A. B., Grove N. J., Butchart A. (2007). Violence: A glossary. Journal of Epidemiology and Community Health.

[B32-behavsci-15-01117] Silverman M. M., Berman A. L., Sanddal N. D., O’carroll P. W., Joiner T. E. (2007a). Rebuilding the tower of babel: A revised nomenclature for the study of suicide and suicidal behaviors. Part 1: Background, rationale, and methodology. Suicide & Life-Threatening Behavior.

[B33-behavsci-15-01117] Silverman M. M., Berman A. L., Sanddal N. D., O’carroll P. W., Joiner T. E. (2007b). Rebuilding the tower of babel: A revised nomenclature for the study of suicide and suicidal behaviors. Part 2: Suicide-related ideations, communications, and behaviors. Suicide & Life-Threatening Behavior.

[B34-behavsci-15-01117] Valdez-Santiago R., Gonzalez-Forteza C., Juarez F., Borges G. (2018a). Suicide attempts among Mexican adolescents: Prevalence and associated factors. Salud Pública de México.

[B35-behavsci-15-01117] Valdez-Santiago R., Híjar M., Rojas-Martínez R., Heredia-Pi I. (2020). Violencia en adolescentes mexicanos: Prevalencia y factores asociados. Salud Pública de México.

[B36-behavsci-15-01117] Valdez-Santiago R., Solórzano E. H., Iñiguez M. M., Burgos L. Á., Gómez Hernández H., Martínez González Á. (2018b). Attempted suicide among adolescents in Mexico: Prevalence and associated factors at the national level. Injury Prevention: Journal of the International Society for Child and Adolescent Injury Prevention.

[B37-behavsci-15-01117] Van Dulmen M., Mata A., Claxton S., Klipfel K., Schinka K., Swahn M., Bossarte R. (2013). Longitudinal associations between violence and suicidality from adolescence into adulthood. Suicide & Life-Threatening Behavior.

[B38-behavsci-15-01117] White R. G., Liu A., Gong E., Heise L. (2024). Intimate partner violence and mental health outcomes in women: A global systematic review and meta-analysis. The Lancet Psychiatry.

[B39-behavsci-15-01117] White S. J., Sin J., Sweeney A., Salisbury T., Wahlich C., Montesinos Guevara C. M., Gillard S., Brett E., Allwright L., Iqbal N., Khan A., Perot C., Marks J., Mantovani N. (2024). Global prevalence and mental health outcomes of intimate partner violence among women: A systematic review and meta-analysis. Trauma, Violence & Abuse.

[B40-behavsci-15-01117] World Health Organisation [WHO] (2004). Violence prevention alliance (VPA): World report on violence and health.

[B41-behavsci-15-01117] World Health Organization [WHO] (2021). Suicide worldwide in 2019: Global health estimates.

